# Detection of Lethal Bronzing Disease in Cabbage Palms (*Sabal palmetto*) Using a Low-Cost Electronic Nose

**DOI:** 10.3390/bios10110188

**Published:** 2020-11-23

**Authors:** Martin J. Oates, Nawaf Abu-Khalaf, Carlos Molina-Cabrera, Antonio Ruiz-Canales, Jose Ramos, Brian W. Bahder

**Affiliations:** 1 Department of Engineering, School of Engineering of Orihuela (EPSO), Miguel Hernández University (UMH), Carretera de Beniel, km 3.2, 03312 Orihuela, Alicante, Spain; moates@btinternet.com (M.J.O.); cmolina@umh.es (C.M.-C.); 2 Department of Agricultural Biotechnology, College of Agricultural Sciences and Technology, Palestine Technical University-Kadoorie (PTUK), P.O. Box 7, Tulkarm, Palestine; n.abukhalaf@ptuk.edu.ps; 3 College of Computing and Engineering, Nova Southeastern University (NSU), 3301 College Avenue, Fort Lauderdale, FL 33314-7796, USA; jr1284@nova.edu; 4 Department of Entomology and Nematology, University of Florida–Fort Lauderdale Research and Education Center, Davie, FL 33314-7719, USA; bbahder@ufl.edu

**Keywords:** electronic nose, plant disease, diagnostics, Discrete Fourier Transform (DFT), Principal Component Analysis (PCA)

## Abstract

Lethal Bronzing Disease (LB) is a disease of palms caused by the 16SrIV-D phytoplasma. A low-cost electronic nose (eNose) prototype was trialed for its detection. It includes an array of eight Taguchi-type (MQ) sensors (MQ135, MQ2, MQ3, MQ4, MQ5, MQ9, MQ7, and MQ8) controlled by an Arduino NANO^®^ microcontroller, using heater voltages that vary sinusoidally over a 2.5 min cycle. Samples of uninfected, early symptomatic, moderate symptomatic, and late symptomatic infected palm leaves of the cabbage palm were processed and analyzed. MQ sensor responses were subjected to a 256 element discrete Fourier transform (DFT), and harmonic component amplitudes were reviewed by principal component analysis (PCA). The experiment was repeated three times, each showing clear evidence of differences in sensor responses between the samples of uninfected leaves and those in the early stages of infection. Within each experiment, four groups of responses were identified, demonstrating the ability of the unit to repeatedly distinguish healthy leaves from diseased ones; however, detection of the severity of infection has not been demonstrated. By selecting appropriate coefficients (here demonstrated with plots of MQ5 Cos1 vs. MQ8 Sin3), it should be possible to build a ruleset classifier to identify healthy and unhealthy samples.

## 1. Introduction

Lethal bronzing disease (LB), previously called Texas Phoenix Palm Decline (TPPD), is a palm disease caused by the 16SrIV-D phytoplasma. This palm disease was observed for the first time within the continental United States, in the state of Texas, in 1978, near the Rio Grande Valley [1]. However, it was not until 2006 when it was found in Florida, on the central western coast, with Hillsborough County as the location of the introduction of the pathogen [2]. In 2009, the LB phytoplasma was isolated for the first time from a native palm, *Sabal palmetto* [3]. In 2011 it was documented in the pygmy date palm (*Phoenix roebelenii*) [4]. Currently, LB has spread throughout most of the state [5]. The disease has been observed in 12 species of palms [6], with *Phoenix canariensis* (Canary Island date palm), *Phoenix dactylifera* (edible date palm), *Phoenix sylvestris* (wild date palm), and *Sabal palmetto* (cabbage palm) being some of the most severely impacted [7]. The symptoms of LB are very similar to lethal yellowing (LY) [8], which includes premature fruit drop/necrosis of the inflorescence, discoloration of the oldest foliage that progresses to younger leaves over time, and finally spear leaf collapse and tree death. Traditionally, validation of infection status of LB infected palms has relied on molecular diagnostics that include quantitative PCR (qPCR) and digital PCR (dPCR) [9,10]. Many of the palm species affected by LB are high value, ornamental species, and given the extent of LB in Florida, economic losses to green industries are in the millions of dollars. Because of the widespread distribution of LB in Florida and the severe economic impact, novel detection tools that allow for rapid screening of large areas will be critical for identifying outbreaks earlier, allowing for management protocols to be implemented to reduce economic losses.

Complementary to the traditional plant disease detection methods, based on molecular techniques, new methods have appeared to be useful tools for plant disease detection, such as spectroscopic and imaging techniques or volatile organic compound (VOC) analysis, where an infected plant is expected to have a different VOCs emission to that of a healthy plant [11]. For VOCs detection purpose, electronic noses have become a convenient device [12]. An electronic nose (eNose) is composed of a multi-sensor array responsible for detecting more than one chemical component [13]. The varied applications of this device are known, commonly used for medical purposes. Medical purposes [14,15,16], food and beverage industry [17,18,19,20], environment monitoring [21,22,23], and explosives [24,25].

The development of technologies, specifically eNose devices, for disease diagnostic applications, has quickly accelerated in the last decade. The impressive rate of technological development of these devices, particularly in the field of disease detection, has been reached to a large extent because of the discovery of new electronic methods and operating mechanisms associated with the chemical detection of complex gas mixtures, which consist mainly of VOCs. The improvement in technologies and sensors, automatic learning such as artificial neuronal networks (ANNs), libraries and disease reference databases, data analysis software, and disease biomarkers identification have also contributed to the advances in the diagnostic methods [26]. These essential advances have brought large, useful eNose applications for detections of diseases elicited by different causes (biotic, abiotic, and genetic), which occur in a variety of living organisms, spanning plants and animals [27].

**Figure 1:**
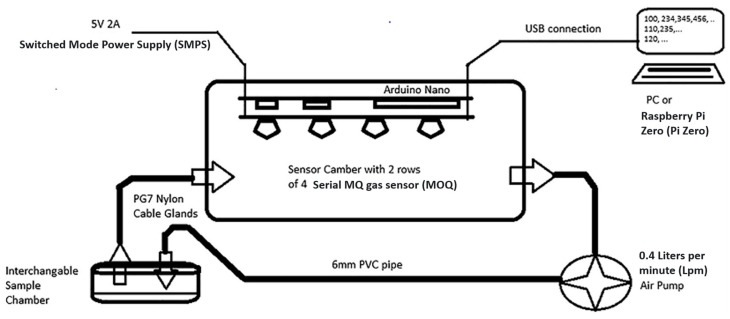
Electronic nose (eNose) system schema [28].

Metal oxide semiconductor sensors (MOS) are one of the most commonly used sensors in electronic noses. Low-cost devices typically employ catalytic Taguchi-type (MQ series) gas sensors [18,28,29,30,31,32] (Figure 1). The MQ series is a family of gas sensors designed for detecting the presence of a variety of chemical components in the air. A large range of MQ sensors is manufactured, with each one designed to be predominantly sensitive to one or a few chemical components, such as alcohol, ammonia, LPG, or methane. MQ sensors must be calibrated before use, and even after calibration, these sensors do not offer the reliability necessary for security purposes. Alternatives could include electrochemical gas sensors and those using specific catalytic pathogen reaction chemicals; however, these were not considered in this research due to their costs typically being two orders of magnitude higher than basic MQ sensors. This would defeat the objective of a low-cost unit.

In this experiment, we aimed to test a low-cost eNose device using an array of eight MQ sensors for detecting LB in palms. These eight sensors were selected due to their low cost, easy availability, wide spectrum of detected gases, and demonstrated ability in other eNose applications [18,28,29,30,31,33,34]. Each sensor is manufactured to detect a set of target gases (MQ2 = LPG, Hydrogen and Propane, MQ3 = Alcohol, MQ4 = Methane, MQ5 = Hydrogen and LPG, MQ7 = Hydrogen and Carbon Monoxide, MQ8 = Hydrogen, MQ9 = Carbon Monoxide and LPG, and MQ135 = Ammonia, Hydrogen Sulfide, and Benzene) as well as other unspecified VOCs; whilst there is apparent overlap in the above list, relative detection rates vary, and most importantly it is not these specific gases that we hope to detect, rather a repeatable “signature” response across the array representing VOCs emitted by unhealthy plants. For this purpose, different leaves of Florida cabbage palm affected by LB in several stages of symptom progression were tested. Captured MQ sensors frequency responses were analyzed by the method of principal component analysis (PCA), finding a good approach to LB identification.

## 2. Materials and Methods

To develop the experiment, several samples of leaves of cabbage palms representing three different stages of symptom progression in four different stages (early, moderate, and late infected) were submitted to analysis by means of a prototype eNose. The main aspect of each material and method are described in the following lines. A healthy specimen with no observable symptoms was included as a control.

### 2.1. Sample Selection and Confirmation of Infection Status

**Figure 2:**
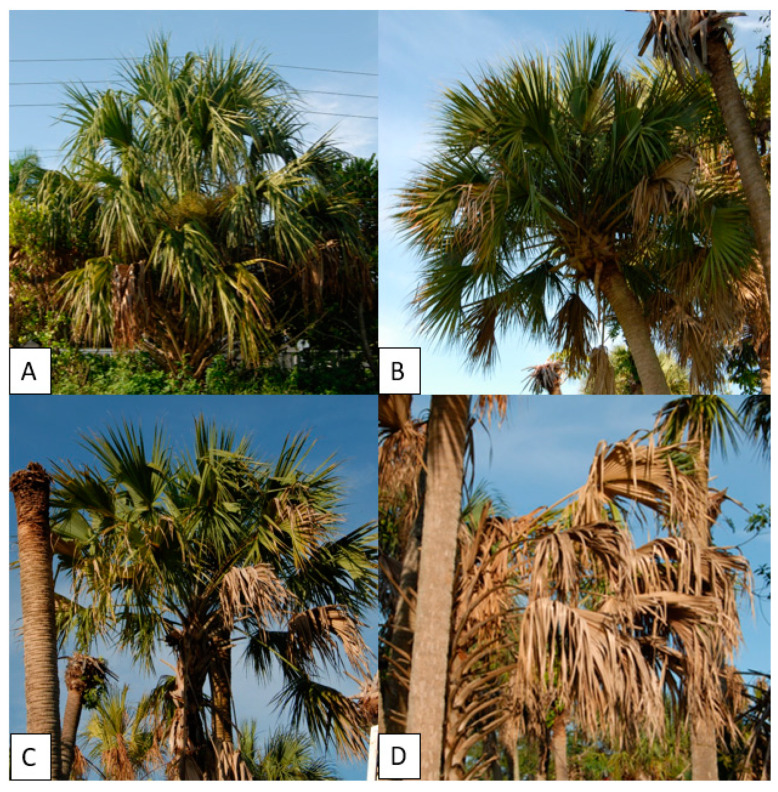
Specimens of Sabal palmetto analyzed for the utility of e-noses for detecting infection by the 16SrIV-D phytoplasma; (**A**) healthy/asymptomatic, (**B**) early symptomatic (ES), (**C**) moderate symptomatic (MS), and (**D**) late symptomatic (LS).

Sample selection of palms used for analysis occurred at the Fort Lauderdale Research and Education Center (FLREC) in Davie, FL, USA. Four specimens of cabbage palm (Sabal palmetto) were selected for analysis; (1) healthy/asymptomatic (HS), (2) LB-infected/early-stage symptoms (ES), (3) LB-infected moderate-stage symptomatic (MS), and (4) LB-infected/late-stage symptomatic (LS) (Figure 2). All palms had their infection status confirmed by quantitative PCR and melt curve analysis. DNA was extracted from the palms by taking 3 g of trunk tissue approximately 1 m above ground level. Tissue was macerated in a BioReba extraction bag with 5 mL guanidine buffer (guanidine thiocyanate—4M, 3M sodium acetate—0.2M, 0.5M EDTA—0.25M, PVP-40—0.0006M) using a HOMEX6 tissue homogenizer. Following maceration, 400 µL of homogenate was transferred to a 2 mL microcentrifuge tube and processed using the DNeasy Plant Mini kit (Qiagen) as per the manufacturer’s instructions. All qPCR assays were performed in 20 µL reactions comprised of 1 µL of DNA template, 50% SsoFast EvaGreen with Low ROX supermix (Bio-Rad, Hercules, CA, USA), 2% polyvinyl pyrrolidone (MW 40,000) (PVP-40), 0.15 µM of each primer with the remaining volume made up with nuclease-free water. Primers used were the PP16S-32 (forward) and PP163 (reverse) [9]. Thermal cycling conditions for qPCR assays were as follows: initial denaturation at 95 °C for 2 min followed by 35 cycles of denaturation at 95 °C for 30 s, annealing at 64 °C for 30 s, and extension at 72 °C for 15 s with endpoint detection, followed by melt curve analysis, comprised of a denaturation step of 95 °C for 30 s, 55 °C for 1 min, and 95 °C for 1 min with continuous detection on the ramp cycle from 55 to 95 °C at 1.3 °C/s ramp speed. All qPCR reactions were run on a StepOnePlus Real-Time PCR System (Applied Biosystems) in triplicates. A confirmed infected cabbage palm isolate from [9] was used as a positive control, a healthy cabbage palm isolated by more than 200 m from the disease area was used as a healthy control, and a water control were also included.

### 2.2. eNose

The device used for this experiment (Figure 1) was composed of a matrix of 8 MQ sensors (MQ2, MQ3, MQ4, MQ5, MQ7, MQ8, MQ9, and MQ135,) with an Arduino NANO^®^ as the data acquisition equipment. Samples were placed into individual 125 mL glass sample chambers, which were connected in turn via 6 mm OD (4 mm ID) PVC pipe to a 700 mL PP5 (Food grade PVC) sensor chamber (containing the MQ sensor array). This was then connected via 4 mm ID PVC pipe to a 0.4 Lpm air pump, which then returned the air to the sample chamber. For fresh air insertion, no sample jar was connected to the system. All the sensors were subjected to circa 48 h burn-in. After the “burn-in”, trimpot resistors (described below) for each sensor were adjusted to get a baseline response of 2 volts under steady-state 5 volts heater conditions. This should be checked and reset after every 200 h of use, and the 48 h “burn-in” repeated if the device is left unused for a period exceeding three months. Voltage cycles ranging from 1.6 V to 5 V were applied sinusoidally to the heaters over a cycle of approximately 2.5 min. This sinusoidal waveform was generated in 256 discrete time steps by the Arduino NANO^®^, which fed an 8 bit PCF8591 digital to analog converter (DAC). The resultant signal was fed to an array of eight L272M operational amplifiers, configured in unity gain mode, one for each sensor heater.

Due to the nominal impedance of each sensor heater being around 32 Ohms, each operational amplifier had to supply up to 160 mA. Each sensor´s sense resistor was connected to ground at one end, and the other end was connected to both an Arduino Nano DAC input channel and via a variable resistor to 5 V. 50 k ohm trimpots were used for this purpose. For MQ3, 100 k ohm trimpot was used instead. Trimpots were adjusted before the experiment to equilibrate the intrinsic sensor variabilities due to the manufacturing process. After 10 ms of a new voltage step output, the microcontroller conducted five consecutive reads on each ADC input channel. The first read was discarded, and the remaining four reads were averaged to reduce noise impact. The result of this operation was sent to a PC logging data system via serial communication at a 112,500 baud rate.

### 2.3. Experiment Condition

**Figure 3:**
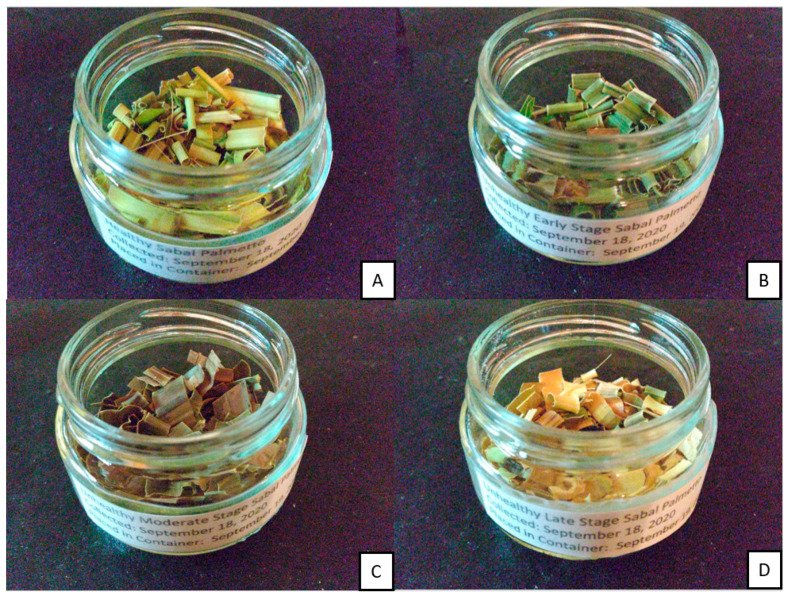
Samples of the Florida Sabal Palmetto. (**A**) healthy/uninfected (HS), (**B**) early-stage (ES), (**C**) moderate-stage (MS), and (**D**) late-stage (LS).

Four samples of cabbage palm were collected and placed in plastic bags. A healthy/uninfected sample (H), along with infected early-stage (ES), infected moderate-stage (MS), and infected late-stage (LS) samples, were collected for further analysis on 18 September 2020. These were then shredded and placed in glass jars, as shown in Figure 3. Inside the glass jars, a 10 mL vial containing Magnesium Sulfate Anhavarous S25414B was placed to control the humidity of the samples. The samples were then placed at 4 °C. The samples were maintained at 4 °C until 26 September 2020. Samples were subsequently heated to 25 °C while the experiments were being conducted. Prior to conducting the experiments, the 10 mL vials were removed. Then after the experiments were concluded, the vials were placed back inside the glass jars for storage.

**Figure 4:**
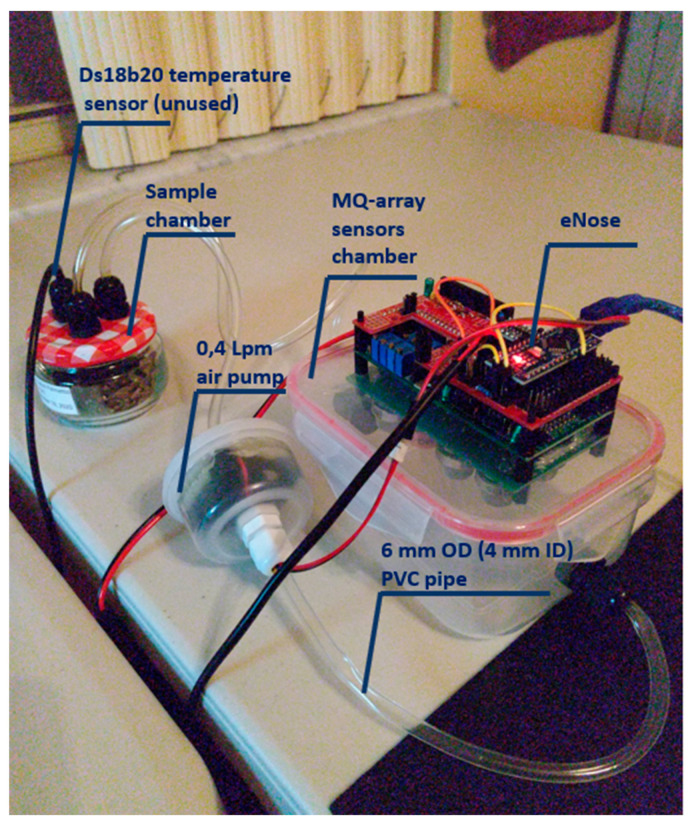
Picture of the electronic nose collecting data. Main parts of the prototype.

Prior to starting the experiments, the electronic nose was left operating overnight with the sampling chamber connected to the air (Figure 3) in order to purge the gas sensors. The experiments consisted of a sequence of nine, 30 min intervals with the samples placed in the eNose sampling chamber, alternating with fresh air. The sequence was: air; H—air—ES—air—MS—air—LS—air. Three consecutive days were used for conducting the experiments: 26–28 September 2020. The experiments lasted four and a half hours each. Figure 4 demonstrates the electronic nose collecting data.

### 2.4. Discrete Fourier Transform (DFT)

As the sensors are exposed to gaseous emission from the sample, their resistance changes [35]. At the same time, the sensor response depends on temperature [36]. As a sinusoidal voltage is applied to the sensor heater, temperature varies, and therefore sensor response is affected. The response is seen as a deformed waveform of the fundamental sinusoidal waveform applied to the heater. Due to the exposure to different gaseous emissions, the sensors offered different deformations regarding the fundamental heater waveform. To quantify this deformation, the results were subjected to a Discrete Fourier Transform (DFT). Sine and cosine coefficients amplitude for increasing harmonics components were analyzed. This technique has been shown to be effective in reducing sensor susceptibility to drift [33] and improves sensitivity and selectivity [36].

**Figure 5:**
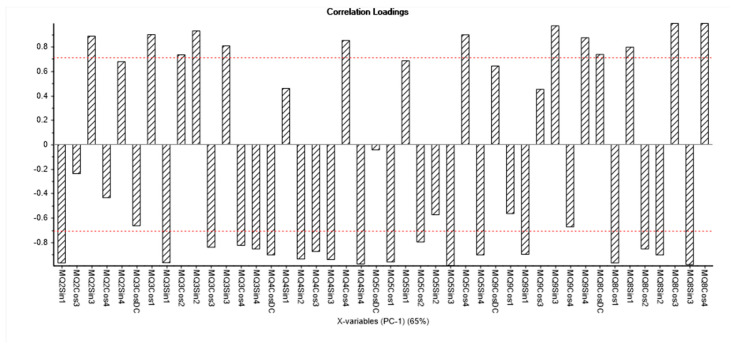
Readings from sensor array (with error bars).

In Figure 5, readings from sensor array (with error bars) for PCA analysis (PC1) are presented (see Section 3). In this loading plot, the most variance was greater than 0.7. The repeatability and reproducibility (R and R) of the sensor array were acceptable (was less than 10%).

A Set of 256 steps was used for performing the Fast Fourier Transform algorithm (FFT). For this, a Matlab (1 Apple Hill DriveNatick, MA, USA) application was developed. This software generated a set of DFT coefficients for each of the 256 readings for each sensor. The coefficients of the first five harmonics were used for the later analysis. As the stronger responses were expected to be found in the first harmonics, hence higher harmonics were dismissed.

### 2.5. Data Analysis

Data from the first, second, and third cycles after a new sample was introduced to the system were excluded from the analysis since this was the time to reach a stable concentration of the palm VOCs in the sensor chamber. Because of the sample exposure periods, typically, 13–14 cycles of 256 steps were applied for the “fresh air” injection, whereas 13–14 cycles were used for sample exposition. The last and first cycles of each sample were considered “transition” and hence dismissed for the analysis. 9th, 10th, 11th, and 12th cycles after sample introduction were chosen for posterior analysis.

Normalized cosine and sine coefficients of the DFT for the first five harmonics were subjected to multivariate data analysis using PCA.

**Figure 6:**
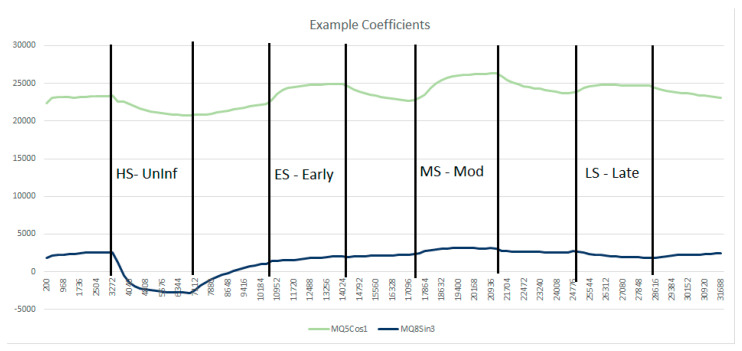
Evolution of MQ5 Cos1 and MQ8 Sin3 during the first experiment.

Figure 6 plots the evolution of two selected Sensor DFT coefficients, demonstrating how these values change during the experiment. Here MQ5 Cos1 (the amplitude of the first cosine harmonic for the MQ5 sensor response) is plotted alongside MQ8 Sin3 (the amplitude of the third sine harmonic for the MQ8 sensor). The MQ5 Cos1 trace clearly shows that an uninfected sample gives a negative response (relative to fresh air), whilst an infected sample gives a positive response.

### 2.6. PCA

Unscrambler Software (Ver. 10.3, Camo, Norway) was used to carry out PCA for 16 samples in each experiment—i.e., healthy/uninfected (HS), early-stage (ES), moderate-stage (MS), and late-stage (LS)—using cosine and sine delay flip-flop (DFF) coefficients. PCA is an unsupervised liner method and is used in many applications for studying the relationship between samples and variables used in research [37,38].

## 3. Results and Discussion

All palms included in the study that presented symptoms (ES, MS, and LS) tested positive for the LB phytoplasma with strong cycle threshold (Ct) values (Table 1) and had melting temperature (Tm) products that matched the positive control isolate of LB (Table 1), confirming that symptomatic palms were infected with the 16SrIV-D phytoplasma. The healthy palm (HS) used in this study tested negative for the presence of the LB phytoplasma (Table 1).

For each experiment, four samples with four replications (i.e., the total analyzed samples are sixteen) were used for building the PCA scores plot, which explains the relationship between samples. Full cross-validation was used for the validation set due to the small number of samples [34].

**Figure 7:**
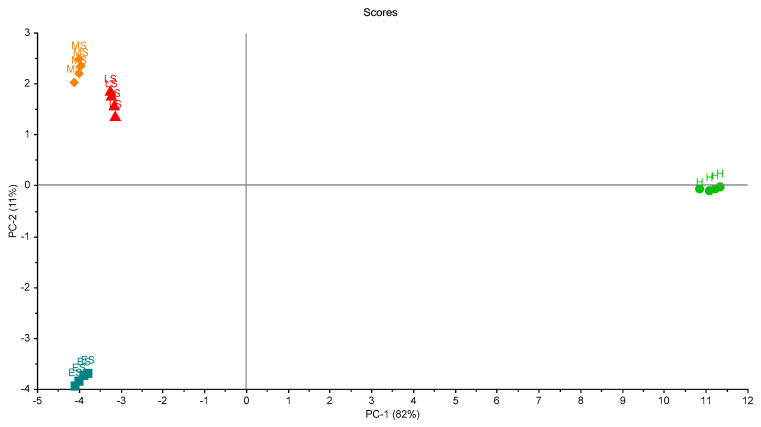
Scores plot of the samples in the first trial of measurements. A clear separation between grouped samples is presented. Healthy samples (H), infected early-stage (ES), infected-moderate-stage (MS), and infected late-stage (LS).

Figure 7 shows the scores plot of the four samples in the first trial. Two principal components (PCs) explained 93% of the total variation of the data. The four groups of palm samples were clearly classified. Moreover, the figure shows that the variation between samples in each group was small, and this indicates the ability of the eNose to make accurate measurements.

**Figure 8:**
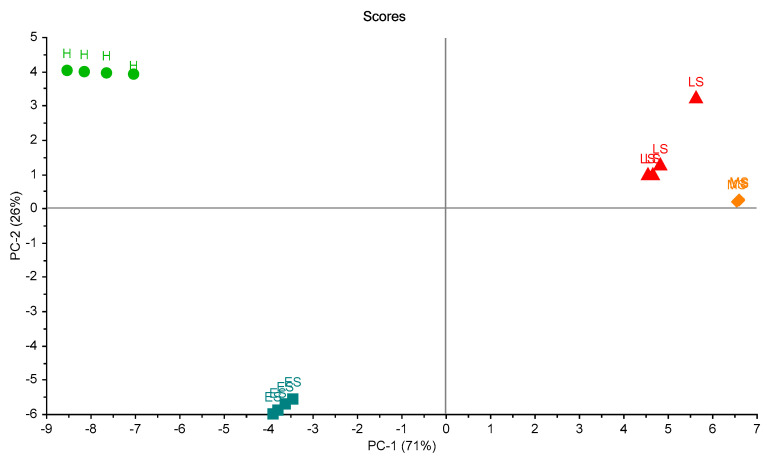
Scores plot of the samples in the second trial of measurements. A clear separation between samples is shown. Healthy sample (H), infected early-stage (ES), infected moderate-stage (MS), and infected late-stage (LS).

For the second trial, the PCA is shown in Figure 8. Two principal components explained 97% of the total variation. A clear grouping was noticed. This experiment was in line with the previous one, and clearly showed the differences between groups.

**Figure 9:**
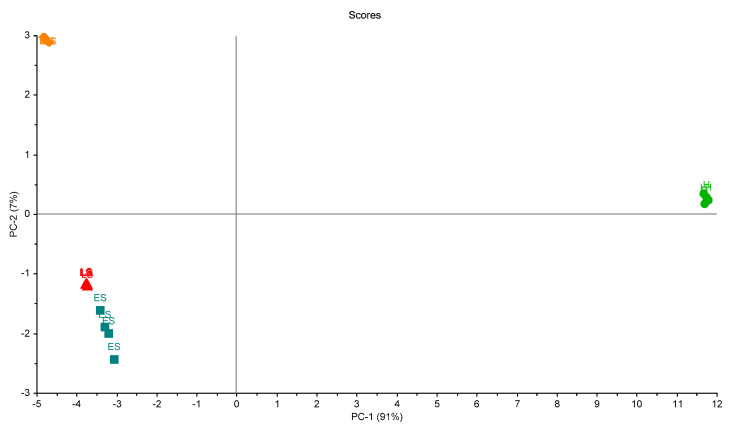
Scores plot of the samples in the third trial of measurements. A clear separation between samples is shown. Healthy sample (H), infected early-stage (ES), infected moderate-stage (MS), and infected late-stage (LS).

The third trial gave a similar response. Figure 9 shows the third trial. Two principal components (2PCs) explained 98% of the total variation. Also, a clear grouping could be seen. The distinction among the four disease stages was clearly obtained with the employed eNose prototype.

**Figure 10:**
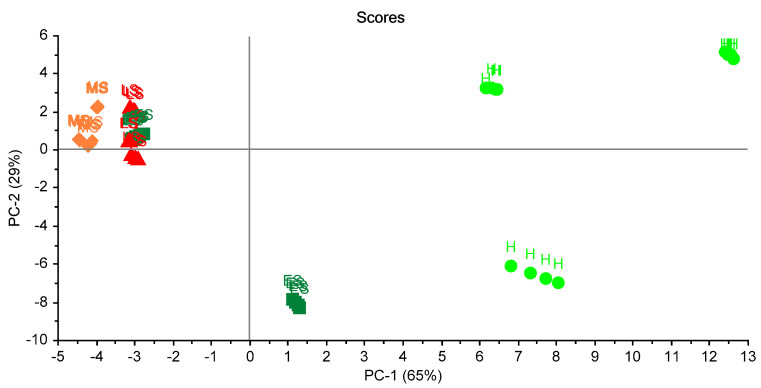
Scores plot of the 16 samples in the three trials. A clear separation between health samples is shown. Healthy sample (H), infected early-stage (ES), infected moderate-stage (MS), and infected late-stage (LS).

For studying the reproducibility of ES in three trials, plotting of PCA scores was carried out (Figure 10). Two principal components (2PCs) explained 94% of the total variation. This figure shows the classification according to the status of the leaves. It can be seen that healthy status (HS) could be classified in a complete way. However, there was a miss classification of other statuses of the leaves.

**Figure 11:**
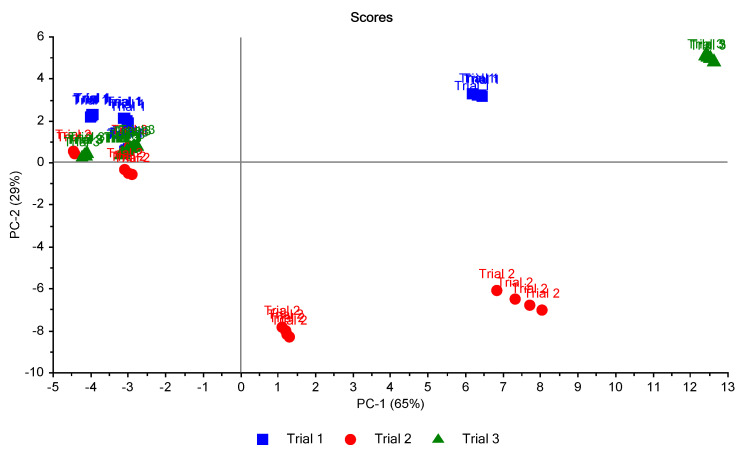
Scores plot of the 16 samples in the three trials classified according to the trials. A clear separation between health samples (on the left) and others (on the right) is shown.

To study the relationship between trials, another plotting of PCA scores was carried out (Figure 11). It was promising to see the healthy leaves (H) and unhealthy early-stage (ES) in trial two could be distinguished in the figure.

**Figure 12:**
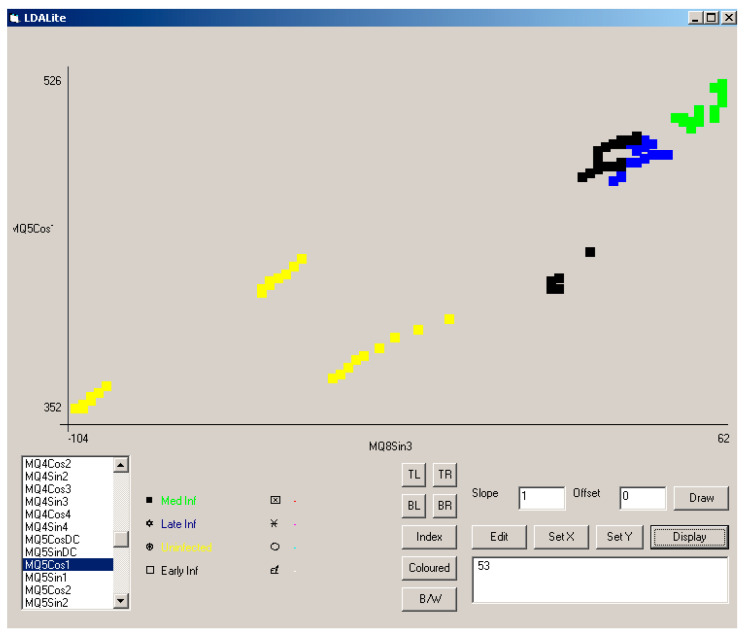
Separation of sample type using MQ5 Cos1 and MQ8 Sin3 across all three experiments. Responses from uninfected (HS) samples are plotted in yellow, from early infection (ES) in black, from moderate infection (MS) in green, and from late infection (LS) in blue. The use of other coefficients can be used to distinguish fresh air, etc.

Whilst the combination of coefficients that make up PC1 and PC2 may be complex, by looking at the responses of selected sensor coefficients, it should be possible to construct a simple ruleset classifier to determine the health of specimens. This is demonstrated in Figure 12, where the amplitude of the first cosine harmonic for the MQ5 sensor response (MQ5 Cos1) was plotted against the amplitude of the third sine harmonic for the MQ8 sensor (MQ8 Sin3) for data from all three experiments. This indicated the grouping of response infection type and showed a clear distinction between infected and uninfected samples. Responses from uninfected (HS) samples are plotted in yellow, from early infection (ES) in black, from moderate infection (MS) in green and from late infection (LS) in blue. The use of other coefficients can be used to distinguish fresh air etc.

As can be seen from Figure 6, Figure 7 and Figure 8, the difference among stages was clearly detected with the used prototype. With the later analysis (Figure 9 and Figure 10) this distinction is not clear. Thus, only the distinction between healthy stage (H) and infected palms (ES, MS, and LS) can be guaranteed with the presented eNose. This may be due to the choice of specific MQ sensors. New trials have to be developed in order to improve the accuracy of the device.

While molecular assays are still the primary means to verify LB infection status in palms, the costs of screening hundreds of palms in a nursery setting can be high. Furthermore, the time and labor to accurately sample and test whole nursery plots is often not possible. Molecular assays require that samples collected in the field be processed and screened in the laboratory. Having a supplemental technology that could allow for rapid identification of palms in the field would allow for more precise sampling of palms in nurseries where palms that register as positive with the eNose could be sampled and tested to confirm and avoid testing large numbers of healthy palms. This would significantly reduce costs to nurseries, providing a positive economic impact on the industry. Another benefit to this technology is its utility in screening palms that are not physically accessible, provided it is adapted to drone technology. There are many stands of naturally occurring cabbage palms in the everglades that serve as important nesting habitat and food sources for local wildlife. Access to these habitats is often both physically and politically impossible or highly impractical, so being able to remotely screen these palms for LB can help assess the risk or impact that LB has on Florida natural habitats, ultimately contributing significantly to conservation efforts.

## 4. Conclusions

This applied technology presented (eNose) has the potential to detect lethal bronzing disease in infected cabbage palms. This is a low-cost technology that has to be improved in order to detect different disease stages. The management software, based in Matlab^®^, can establish the different patterns of specific MQ gas sensors (VOCs detection) in a matrix of eight sensors. A fast Fourier transform algorithm (FFT), with the corresponding sinusoidal and cosine coefficients, was used for obtaining the patterns of each stage in each MQ gas sensor. The employed prototype could be enhanced by adding the statistical analysis into the management software. Moreover, new experiments have to be developed in order to obtain a better detection of each disease stage.

Some of the advantages of this device are as follows:

- The use of low-cost sensors solution is open and allows connection to other devices with standardized outputs on the market.
- It is possible to differentiate infected palms and healthy palms.
- The developed software could be enhanced to include a data server that responds to requests from a data cloud platform or other devices such as mobile phones, tablets, etc.
- The stored data (historical and real-time) can be deposited on dashboards accessible by the web on any browser and are very intuitive to interpret.
- This device is a suitable solution for quick detection of this disease after a ‘training’ period of the device.

With each experiment, four groups were classified perfectly in the three trials. For the possible reproducibility of the experiment, it was possible to distinguish healthy leaves from diseased ones, but it was difficult to distinguish the three stages. Following these preliminary trials, it may be necessary to replace some sensors in the array in order to improve the accuracy of the device and the distinction of stages in the future. Furthermore, additional studies are needed in other species of palm to see if the trends observed in cabbage palm are consistent as well as to test against palms suffering from fungal pathogens.

## Figures and Tables

**Table 1:** qPCR results confirming the infection status of experimental *Sabal palmetto* palms used for eNose analysis.

Sample	Ct	Tm
Healthy/asymptomatic palm (HS)	No Ct	62.0 ± 0.2
Early symptomatic palm (ES)	22.2 ± 0.3	79.8 ± 0.1
Moderate symptomatic palm (MS)	22.9 ± 0.4	79.8 ± 0.1
Late symptomatic palm (LS)	24.1 ± 0.3	79.8 ± 0.0
16SrIV-D positive control	21.9 ± 0.2	79.8 ± 0.0
Healthy control	No Ct	60.0 ± 0.2
Water control	No Ct	54.5 ± 0.2

Ct: cycle threshold.

## References

[1] McCoy R.E. (1980). Lethal Decline of Phoenix Palms in Texas Associated with Mycoplasmalike Organisms. Plant Dis..

[2] Harrison N.A., Helmick E.E., Elliott M.L. (2008). Lethal yellowing-type diseases of palms associated with phytoplasmas newly identified in Florida, USA. Ann. Appl. Biol..

[3] Harrison N.A., Helmick E.E., Elliott M.L. (2009). First report of a phytoplasma-associated lethal decline of Sabal palmetto in Florida, USA. Plant Pathol..

[4] Jeyaprakash A., Sutton B.D., Halbert S.E., Schubert T.S. (2011). First Report of a 16SrIV-D Phytoplasma Associated with Texas Phoenix Palm Decline on Pigmy Date Palm ( Phoenix roebelenii ) in Florida. Plant Dis..

[5] Bahder B.W., Helmick E.E., Mou D.F., Harrison N.A., Davis R. (2018). Digital PCR technology for detection of palm-infecting phytoplasmas belonging to group 16SrIV that occur in Florida. Plant Dis..

[6] Bahder B.W., Soto N., Mou D.F., Humphries A.R., Helmick E.E. (2020). Quantification and distribution of the 16SrIV-D phytoplasma in the wild date palm, phoenix sylvestris, at different stages of decline using quantitative PCR (qPCR) Analysis. Plant Dis..

[7] Harrison N., Elliott M. (2009). Texas Phoenix Palm Decline. Circular PP-243.

[8] Maramorosch K., Hunt P. (1981). LETHAL YELLOWING DISEASE OF COCONUT AND OTHER PALMS. Mycoplasma Diseases of Trees and Shrubs.

[9] Bahder B.W., Helmick E.E., Harrison N.A. (2017). Detecting and differentiating phytoplasmas belonging to subgroups 16SrIV-A and 16SrIV-D associated with lethal declines of palms in florida using qPCR and high-resolution melt analysis (HRMA). Plant Dis..

[10] Bahder B.W., Soto N., Komondy L., Mou D.F., Humphries A.R., Helmick E.E. (2019). Detection and quantification of the 16SrIV-D phytoplasma in leaf tissue of common ornamental palm species in florida using qPCR and dPCR. Plant Dis..

[11] Sankaran S., Mishra A., Ehsani R., Davis C. (2010). A review of advanced techniques for detecting plant diseases. Comput. Electron. Agric..

[12] Wilson A.D. (2020). Noninvasive early disease diagnosis by electronic-nose and related VOC-detection devices. Biosensors.

[13] Karakaya D., Ulucan O., Turkan M. (2020). Electronic Nose and Its Applications: A Survey. Int. J. Autom. Comput..

[14] Zhan X., Wang Z., Yang M., Luo Z., Wang Y., Li G. (2020). An electronic nose-based assistive diagnostic prototype for lung cancer detection with conformal prediction. Meas. J. Int. Meas. Confed..

[15] Matsumoto K., Murakami Y., Shimizu Y., Hirayama T., Ishikawa W., Iwamura M. (2020). Electronic nose to distinguish bladder cancer by urinary odour feature: A pilot study. Cancer Biomark..

[16] Esfahani S., Wicaksono A., Mozdiak E., Arasaradnam R.P., Covington J.A. (2018). Non-invasive diagnosis of diabetes by volatile organic compounds in urine using FAIMs and FOX4000 electronic nose. Biosensors.

[17] Sanaeifar A., Mohtasebi S.S., Ghasemi-Varnamkhasti M., Ahmadi H., Lozano J. (2014). Development and application of a new low cost electronic nose for the ripeness monitoring of banana using computational techniques (PCA, LDA, SIMCA, and SVM). Czech J. Food Sci..

[18] Gonzalez Viejo C., Fuentes S., Godbole A., Widdicombe B., Unnithan R.R. (2020). Development of a low-cost e-nose to assess aroma profiles: An artificial intelligence application to assess beer quality. Sens. Actuators B Chem..

[19] Giungato P., Laiola E., Nicolardi V. (2017). Evaluation of Industrial Roasting Degree of Coffee Beans by Using an Electronic Nose and a Stepwise Backward Selection of Predictors. Food Anal. Methods.

[20] Konduru T., Rains G.C., Li C. (2015). A customized metal oxide semiconductor-based gas sensor array for onion quality evaluation: System development and characterization. Sensors.

[21] Wilson A.D. (2012). Review of Electronic-nose Technologies and Algorithms to Detect Hazardous Chemicals in the Environment. Procedia Technol..

[22] Herrero J.L., Lozano J., Santos J.P., Suárez J.I. (2016). On-line classification of pollutants in water using wireless portable electronic noses. Chemosphere.

[23] Lozano J., Santos J.P., Suárez J.I., Arroyo P., Herrero J.L., Martín A. (2014). Detection of pollutants in water samples with a wireless hand-held e-nose. Procedia Engineering.

[24] Torres-Tello J., Guaman A.V., Ko S.-B. (2020). Improving the Detection of Explosives in a MOX Chemical Sensors Array with LSTM Networks. IEEE Sens. J..

[25] Ratchapakorn N., Ariyakul Y. Development of a Low-cost Explosive Vapor Detector Using Metal Oxide Gas Sensors. Proceedings of the ICSEC 2017—21st International Computer Science and Engineering Conference.

[26] Dan Wilson A. (2017). Annals of Clinical Case Reports Electronic-nose Devices-Potential for Noninvasive Early Disease-Detection Applications OPEN ACCESS. Ann. Clin. Case Rep..

[27] Maniscalco M., Motta A. (2018). Clinical and Inflammatory Phenotyping: Can Electronic Nose and NMR-based Metabolomics Work at the Bedside?. Arch. Med. Res..

[28] Macías Macías M., Agudo J.E., García Manso A., García Orellana C.J., González Velasco H.M., Gallardo Caballero R.A. (2013). Compact and low cost electronic nose for aroma detection. Sensors.

[29] Majchrzak T., Wojnowski W., Dymerski T., Gebicki J., Namiesnik J. (2018). Electronic noses in classification and quality control of edible oils: A review. Food Chem..

[30] Tang K., Chiu S., Pan C., Hsieh H., Liang Y., Liu S. (2010). Development of a portable electronic nose system for the detection and classification of fruity odors. Sensors.

[31] Trirongjitmoah S., Juengmunkong Z., Srikulnath K., Somboon P. (2015). Classification of garlic cultivars using an electronic nose. Comput. Electron. Agric..

[32] Chansongkram W., Nimsuk N. (2016). Development of a wireless electronic nose capable of measuring odors both in open and closed systems. Proc. Comput. Sci..

[33] Oates M.J., Fox P., Sanchez-Rodriguez L., Carbonell-Barrachina Á.A., Ruiz-Canales A. (2018). DFT based classification of olive oil type using a sinusoidally heated, low cost electronic nose. Comput. Electron. Agric..

[34] Zaid A., Abu-Khalaf N., Mudalal S., Petracci M. (2020). Differentiation between Normal and White Striped Turkey Breasts by Visible/Near Infrared Spectroscopy and Multivariate Data Analysis. Food Sci. Anim. Resour..

[35] Nagy A.S., Polanco Risquet A., Martínez de la Cotera O.L., Carralero Ibargollen O. (2020). Medición simultánea de gases con sensores MQ. Ing. Electrónica Automática Comun..

[36] Gajdosik L. (2014). The derivation of the electrical conductance/temperature dependency for tin dioxide gas sensor. Adv. Electr. Electron. Eng..

[37] Abu-Khalaf N., Hmidat M. (2020). Visible/Near Infrared (VIS/NIR) spectroscopy as an optical sensor for evaluating olive oil quality. Comput. Electron. Agric..

[38] Taha H., Nawaf Abu-Khalaf N. Quality control for herbal medicinal plants using a sensor array (an electronic tongue). Proceedings of the 7th International Conference of Biotechnology, Environment and Engineering Sciences (ICBE7).

